# Macroeconomic and Social Indicators to Launch the PM-Based VBHC Model in the Healthcare System in Poland

**DOI:** 10.3390/ijerph19031712

**Published:** 2022-02-02

**Authors:** Ewelina Nojszewska, Agata Sielska

**Affiliations:** Department of Applied Economics, SGH Warsaw School of Economics, 02-554 Warsaw, Poland; asiels@sgh.waw.pl

**Keywords:** value-based healthcare, personalised medicine, KPIs, economic efficiency, clinical effectiveness, DEMATEL

## Abstract

Global health systems face shortages of resources and, above all, money, with a simultaneous increase in health spending, as well as doubts about their effectiveness. In addition, there is a growing sense of greater achievement of the essential goal of clinical effectiveness. In the face of these problems, many centres are working on a new system of financing healthcare providers, primarily hospitals, which provide the most expensive medical services. In the opinion of the authors, an essential element for the implementation of VBHC is a comprehensive knowledge of hospitals, health care, the economy, public finances and the behaviour of members of society, based on KPIs. The work on these is not well advanced, and it seems that without the knowledge of economic and social determinants, it will not be possible to implement an affective VBHC model. Therefore, in the present article, after presenting the current state of research regarding the VBHC, personalised medicine as a prerequisite for achieving clinical effectiveness, and KPIs as a prerequisite for achieving economic efficiency, the authors focus on economic and sociological KPIs. The knowledge gained from this study is necessary to make effective decisions for the appropriate operation of healthcare as a system, and of hospitals in particular.

## 1. Introduction

Healthcare systems in OECD member countries have had serious problems with the efficient use of resources for years. Much of the expenditure on health is ineffective or even wasted. One patient in ten is inappropriately treated, which leads to undesirable effects, i.e., 10% of hospitals’ expenses. Excessive prescription of antibiotics is a particular problem, which gives rise not only to unnecessary costs but also to the resistance to antibiotics. The OECD report shows that it is in line with the estimates that 20% of health expenditure is wasted [1]. Furthermore, in all European countries there is a problem of unsatisfied health needs, which is currently one of the challenges faced by EU member countries [2]. One of the most important proposals to solve this problem is using the resources at hand to meet health needs to achieve clinical effectiveness, together with the fulfillment of the condition of economic efficiency [3]. The development of medical science is costly, and this problem has become a financial challenge to European healthcare systems. It is obvious that a long-lasting growth of spending on health cannot be continued indefinitely [4].

It can be seen in Poland, as well as in many other countries, that there is no convincing approach to the ineffective operation of healthcare systems and no willingness to change the situation. Severe budget constraints and shortages of money are increasingly conspicuous. The increase in spending caused by ageing societies and chronic diseases, as well as higher costs of medical innovation and patient awareness, have been exacerbated by the COVID-19 disaster. During the pandemic, everyone feels there is an urgent need to improve the operation of the healthcare system, which, as all concerned agree, is broken. What society badly needs in this dire situation is a national strategy which creates a model of a clinically effective and economically efficient system, as well as a path to reach it. This target system has to be patient-oriented, which means the maximisation of health output per every euro spent. Efficiently achieved good health results are the real, sole purpose, and not the false savings achieved by limited access to health services and deterioration of their quality. Porter proposed a solution to this problem in the form of a value-based healthcare system (VBHC) [5]. Furthermore, work on this issue has already been undertaken by other centres: the Economists Intelligence Unit, the World Economic Forum with the Boston Consulting Group and the European Commission.

Taking into account the problems with the effectiveness of health care, in the present article, attention has been focused on the model enabling its improvement.

The aim of the article is to present the importance of knowledge obtained thanks to KPIs—using the DEMATEL (Decision-Making Trial and Evaluation Laboratory) tool—for making optimal decisions for the functioning of health care. As numerical data are not always available for the analysis of the determinants of decisions in health care, the DEMATEL tool was chosen. Thanks to the use of this tool, opinions and expertise can be used for quantitative analysis. In this way, the obtained results improve the basis for making decisions in health care, which is of particular importance in the face of resource scarcity. An additional goal is to present an exemplary analysis of economic data for Poland in order to create the fullest possible background for the decisions made in health care.

A condition necessary to implement the VBHC is the use of personalised medicine (PM) mainly in oncology, but also in other selected fields of medicine. While there is no universally accepted definition, the European Union (EU) Health Minister defined it as: *A medical model using characterization of individuals’ phenotypes and genotypes (e.g., molecular profiling, medical imaging, lifestyle data) for tailoring the right therapeutic strategy for the right person at the right time, and/or to determine the predisposition to disease and/or to deliver timely and targeted prevention* [6]. It is commonly believed that health outcomes cannot be maximised without PM-based patient diagnosis and treatment.

In order to implement the VBHC system, it is necessary to formulate key performance indicators (KPIs), which are groups of the most important factors affecting healthcare operation. They enable monitoring, assessing and managing health systems to achieve clinical effectiveness, economic and financial efficiency and availability, as well as equity and quality of treatment. A good KPI should be well-defined, quantifiable, thoroughly communicative and crucial to achieving strategic goals. A famous quote attributed to Florence Nightingale best describes the performance–quality–management relationship: *The ultimate goal is to manage quality. But you cannot manage it until you have a way to measure it, and you cannot measure it until you can monitor it* [7].

**Figure 1:**
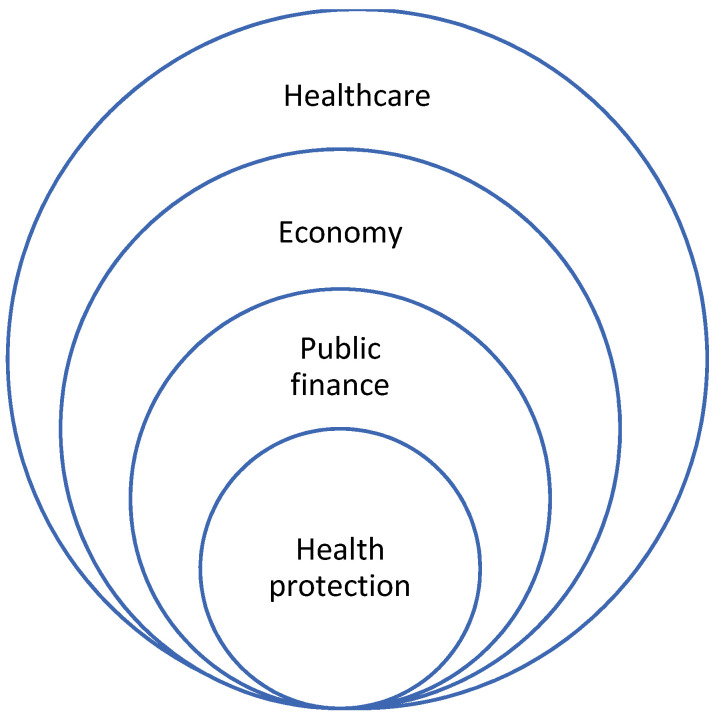
Spheres of impact on healthcare operation. Source: authors’ own material.

It should be emphasised that the operation of hospitals and healthcare providers in general, which translates into the quality and availability of health services, depends on all of the determinants related to this operation. In addition to the organisation, management and financing of healthcare itself, one should have knowledge based on indicators on the state of the economy, public finances and the ways of making decisions, by each member of the society. Thus, indicators related to these areas should be constructed and used. Their impact on health protection is shown in Figure 1 below.

The literature gives the impression that the interest has primarily focused on hospital KPIs. It is obvious to link the operation of hospitals with the performance of healthcare as a whole system. According to the authors, the analysis of the quality and availability of health services cannot ignore the economic situation, which also determines the quality of the state budget and the budgets of local governments; it is also about health spending to be included in the budgets of various ministries or sectors of the economy. All stakeholders belong to a society with a specific culture and mentality resulting from the history of the country and nation. Therefore, their behaviours, primarily in the health protection area, should be a source of knowledge in the creation of all healthcare policies. One may get the impression that a particular challenge requires a sociological approach, thanks to which it will be easier for decision makers to plan each activity to enable an effective implementation of the VBHC.

In the present article, the authors propose a holistic approach, and they focus on the most important aspects, i.e., the PM-based VBHC concept in selected fields of medicine, as well as on KPIs providing knowledge of comprehensive impacts on the quality and availability of health services, which is presented in Figure 1.

### 1.1. Value-Based Health Care (VBHC)

Michael Porter, together with Elizabeth Teisberg [5], created the VBHC concept. They proposed a new approach to organisation, financing and management in healthcare, as the existing healthcare system undermines the efficiency, availability and quality of results. Thanks to their careful analysis of the problems paralysing the operation of healthcare, they formulated and recommended a new approach to the necessary changes. Their analysis shows that stakeholders have competed mainly to shift costs, accumulate bargaining power, and restrict services, rather than create value for patients. According to the authors, this competition is not where it should be. Thus, healthy competition should take place where it means the most, i.e., in the diagnosis, treatment, and prevention of specific health conditions. They created the framework for redefining health care and for moving to a value-based healthcare that advances through the full cycle of care. In the subsequent articles, Porter developed his approach and refined certain aspects of his VBHC concept, i.e., the measurement of improvement in health, innovation slowdown, ill-advised cost containment, and micromanagement of physician practices. He also stressed that the measurement of value will enable the reform of the reimbursement system, the purpose of which is to provide bundled payments covering the full cycle of care (in the case of chronic diseases to cover at least annual periods) [8].

The authors of another article emphasised the possibility of distinguishing key success factors for payers [9]. They noted that although each market is unique, certain strategies will be relevant in all markets for payers which seek to inform and align providers. According to them, the first strategy is investing in data analytical capabilities; the second is to translate insights into compelling materials; and the third is to build trust with providers and patients through the following activities: a focus on value, not cost; patient-centered levers and provider-centered levers.

The World Economic Forum and the Boston Consulting Group (BCG) launched a special project devoted to Value in Healthcare in 2016. It had four basic goals: *To develop a comprehensive understanding of the key components of value-based health systems; to draw general lessons about the effective implementation of value-based healthcare by codifying best practice at leading healthcare institutions around the world; to identify the potential obstacles preventing health systems from delivering better outcomes that matter to patients, and at lower cost; and to define priorities for industry stakeholders to accelerate the adoption of value-based models for delivering care* [10].

Health is considered to be the most important value for enabling a happy and a fulfilling life worldwide. Therefore, healthcare systems tend to provide health to the whole society, and equitable achievement of health for the population reflects the concept of solidarity, which is deeply rooted in European history. In line with the approach of some experts, the concept of value in the context of healthcare is defined as health outcomes relative to monetized inputs, aiming at increasing cost-effectiveness. This interpretation of value is perceived by European Commission experts as too narrow. They argue that the notion of value-based healthcare seems more suitable, and propose to define VBHC as *a comprehensive concept built on four value-pillars: appropriate care to achieve patients’ personal goals (personal value), achievement of best possible outcomes with available resources (technical value), equitable resource distribution across all patient groups (allocative value) and contribution of healthcare to social participation and connectedness (societal value)* [11].

VBHC is a delivery model in which all providers are paid based on patient health outcomes. It differs from the models that operate today, in which providers are paid based on the number of services they deliver. The benefits of such a system are achieved by the entire society and the economy, and in particular by patients, providers and payers. The benefits that extend to all stakeholders mean that: 1/patients spend less money to achieve better health; 2/providers achieve economic/financial efficiency and clinical effectiveness; 3/payers control costs, reduce risk and align prices with patient outcomes; 4/society becomes healthier while reducing overall healthcare spending; 5/the economy achieves faster economic growth and development, which improve the welfare of society.

### 1.2. Personalised Medicine (PM)

The introduction of the VBHC concept into a healthcare system is impossible without relying on personalised medicine, because only then it is possible to further approach clinical effectiveness, which is a condition for achieving financial and economic efficiency. Medical reports show percentages of the patient population for which a particular drug in a class is ineffective, i.e., on average, anti-depressants—38%, asthma drugs—40%, diabetes drugs—43%, arthritis drugs—50%, Alzheimer’s drugs—70%, cancer drugs—75% [12]. Using personalised medicine means that the right drug and the right dosage are selected based on the patient’s genome. This allows a patient to avoid taking a drug that does not work or causes adverse side effects. PM opens up additional possibilities because it reveals the molecular predisposition of each patient to a specific disease, and it enables the application of a proper prevention measure at the right time [13].

The development of personalised medicine depends on interdisciplinary studies, the outcome of which will enable its application in healthcare systems. Two research areas are most important: I—The first group of problems is related to costs and cost-effectiveness, and the following issues must be resolved: *1*/ *how to calculate cost-effectiveness for personalised medicine; 2/ how precision medicine can be cost-effective, maybe even more cost-effective than traditional approaches; 3/ how to introduce flexibility in conventional payment systems to account for performance (outcome-based payments);* II—The second group of problems is connected with innovative financing and payment systems: *1/ cost and pricing: how to calculate the price of a unique life-time dose for an inherently individualised cure; 2/ how to develop new payment systems such as those that are widely used in other fields affected by typically low-probability/high-impact events (e.g., loans, mortgages, securitisation); 3/ how to make these systems affordable and socially acceptable; 4/ how to establish the performance of an individualised treatment and how to modulate the price in relation to its outcome or effectiveness and overall value; 5/ how to introduce planned flexibility; 6/ the cost of “curative” initial treatments may be at the expense of payers who are not the ones who will see the benefit in the long-term (e.g., Alzheimer’s disease)* [14].

To summarise, it can be said that thanks to PM, it is possible to: 1/ assess risk thanks to genetic tests; 2/ use prevention measures; 3/ detect diseases in their earliest stages; 4/ diagnose on an individualised basis; 5/ treat in targeted way; 6/ control treatment and its effects. The use of VBHC and the diagnostic possibilities of PM make us aware of the necessity to use health economics, because it allows for the determination value-for-money. Thus, PM promises to lower healthcare costs through early detection, prevention, accurate risk assessments, and also effectiveness in care delivery. It is of special importance because these all have a significant impact on economies and societies.

### 1.3. Key Performance Indicators (KPIs)

Striving to implement a PM-based VBHC, it should be emphasised that this will be possible not only thanks to the progress in medical science, but also thanks to the introduction of appropriate incentives for health protection, among which the most important are KPIs.

Thanks to personalised medicine, it is known what the result of therapy will be, which means that healthcare will cease to be the so-called “experience good”, the quality of which can be known after consuming it, and will become the so-called “search good”, the quality of which is known even before therapy, albeit in an imperfect way [15]. Healthcare decision makers want to understand the economic value of both PM and optimal pricing of all resources. One of the challenges faced by PM is the issue of the adherence to treatment discipline on the part of patients. Research shows that non-adherence is an important source of loss. For example, in US healthcare, losses caused by this reached about 2.3% of the GDP in 2014 [16].

The possibility of implementation of the PM based VBHC approach depends on the multi-dimensional analysis of organisation, financing and management of the healthcare system as a whole, as well as all providers, payers and patients. However, it is not enough to gain knowledge of the healthcare sector only. It is also indispensable to know at least the economic and social determinants. The most important tool to acquire the necessary knowledge is KPIs. It should be noted that it is not only about KPIs related to the medical effectiveness and efficiency of the healthcare system and all stakeholders. These types of KPIs have long been created and their values are well known. KPIs showing macroeconomic determinants, public finances, social behaviour and the way decisions are made by politicians, bureaucrats, individuals and all of society are no less important.

It has become a goal for decisionmakers and researchers to develop a group of strategic KPIs to monitor and improve the performance of healthcare systems and all stakeholders, especially hospitals. Such KPIs require an operational definition, since they are, in fact, quantitative measures of quality [17,18,19]. It should be mentioned that many hospitals have been developing KPIs for monitoring, measuring, and managing their performance. Managers want to ensure the achievement of clinical effectiveness and economic efficiency, and also equality and quality of health services [20]. Examples of KPIs used in hospitals include *1/ Average Hospital Stay: evaluate the amount of time patients are staying; 2/ Bed Occupancy Rate: monitor the availability of hospital beds; 3/ Medical Equipment Utilisation: track the utilisation of your equipment; 4/ Patient Drug Cost Per Stay: improve cost management of medication; 5/ Treatment Costs: calculate how much a patient costs to your facility; 6/ Patient Room Turnover Rate: balance the turnover with speed and quality; 7/ Patient Follow-up Rate: measure the care for your patients over time; 8/ Hospital Readmission Rates: track how many patients come back; 9/ Patient Wait Time: monitor waiting times to increase patient satisfaction; 10/ Patient Satisfaction: analyse patient satisfaction in detail; 11/ Staff-to-Patient Ratio: ensure you have enough staff to care for patients; 12/ Canceled/missed appointments: keep track of patients’ appointments; 13/ Patient Safety: prevent incidents happening in your facility; 14/ ER Wait Time: identify rush hours in your emergency room; 15/ Costs by Payer: identify the type of health insurance of your patients* [21].

Researchers want to construct the most important (economic and social) classes of KPIs and all individual indicators belonging to them. To achieve this, they have to answer a number of questions which define the framework for the effective performance of healthcare and all stakeholders. It is imperative to know and understand all the economic and social determinants of performance frameworks for healthcare, its economic efficiency and clinical effectiveness [22].

As there are attempts to implement PM, which is a condition for the use of VBHC, in European Union member states, the creation of KPIs is of particular importance. Therefore, this article attempts to create two types of KPIs for Poland—macroeconomic and social—using the DEMATEL method. These are to enable the determination of the amount of money available for treatment in the public and private healthcare segments in Poland.

Continuing the approach used in the literature, in the present article, the authors focus their attention on macroeconomic KPIs determining the revenues of public healthcare, as well as on social indicators determining the revenues of private healthcare. In the next step, they determine these two types of indicators for Poland. In order to achieve this, the rest of the paper is organised as follows: first, the structure of the study is presented, with particular emphasis on the relationships among the social and economic KPI variables, and then all types of calculated results are described in detail and discussed.

## 2. Materials and Methods

### 2.1. Study Design

Due to the fact that it is basically impossible to determine the optimal level of the values of economic indicators, a relative point of view was adopted in the design of the indicator, in which its values were considered in relation to a certain reference point (benchmark). In the text, we propose three benchmarks; however, it is not a closed set and it is possible to determine others, depending on the available data as well as the perspective taken into account in the assessment.

In the article, the DEMATEL tool was chosen to conduct the analysis, because it allows the use of opinions, assessments, and expert opinions for quantitative analysis, when there are no numerical data. Thanks to this, the knowledge about the determinants of the functioning of health care is deepened, which is conducive to making the most effective decisions. The knowledge was obtained by quantitative analysis based on numerical data and based on the opinions of experts. Furthermore, this method was chosen over other multicriteria methods because it allows the analysis of a complex system ‘disentangling’ cause–effect relationships. In our opinion it is an important feature in analyzing economic problems.

In the construction of the proposed economic and social indicators, we suggest the following procedure:

1. Conversion of variables into stimulants;
2. Assessment of relationships among the indicator variables (DEMATEL method);
3. Assessment of the relative significance of the KPI variables;
4. Determination of the weights of the KPI variables;
5. Benchmark determination;
6. Calculation of the indicator in relation to a selected benchmark.

The data for the construction of social KPIs may be collected from surveys. Since the question asked to respondents about their assessment of their chosen sphere of life in previous years may be fraught with errors, higher frequency data, e.g., monthly, can be used to assess social KPIs. However, due to fact that this text was written during the COVID-19 pandemic, no surveys were conducted and the steps discussed below are based on the economic KPI, because the authors had a set of objective data from recent years for it.

Step 1. Conversion of Variables into Stimulants

The 5 selected variables are different in character. The level of wages and GDP are stimulants (higher values are desirable), the unemployment rate and the budget deficit are destimulants (lower values are desirable), and the level of inflation is a nominant (values within a certain fixed range are desirable; in our study, we assumed that the inflation target was an optimal level in this case).

The conversion of the unemployment rate and the budget deficit into a stimulant took place according to the formula:

$$x_{i}^{\prime }=\frac{\underset{i}{\min}x_{i}}{x_{i}}$$

where: $x_{i}—$initial value of the variable,

$x_{i}^{\prime }$—value of a variable converted into a stimulant.

We assumed that the above equation was well-defined due to the fact that full employment is basically unattainable in the economy.

The conversion of the inflation rate into a stimulant occured according to the formula:

$$x_{i}^{\prime }=\{\begin{array}{ccc} x_{i}/ci & for & x_{i}\leq ci\\ ci/x_{i} & for & x_{i}>ci \end{array}$$

where:

$x_{i}—$initial value of the variable,

ci—inflation target,

$x_{i}^{\prime }$—value of a variable converted to a stimulant.

In the case of variables that were already stimulants in character, we took $x_{i}^{\prime }=x_{i}$. Since KPIs are based on a relative approach in which the reference point is a different value (maximum, minimum or precedent), there was no need to normalise the values taken by these variables.

Step 2: Assessment of Relationships among the Indicator Variables (DEMATEL Method)

The decision-making trial and evaluation laboratory method (DEMATEL) was used to assess relationships among the KPI variables; this was the decision support method developed in 1973 by Gabus and Fontela [23]. It allows determination of the cause-and-effect interdependencies between objects in complex systems [24]. It is used to identify relationships among performance indicators and to determine KPIs in problems related to, for example, university [25] or hospital [26] management.

The starting point in the DEMATEL method is so-called impact matrix *B*, whose elements $b_{ij}$ denote the impact of variable *i* on variable *j*. The force of impact is determined by positive integers, with 0 indicating no impact.

The values of matrix *B* are then normalised according to the formula

$$BN=\frac{1}{\lambda }\cdot B$$

where:

$$\lambda =\max\left\{\max\left\{\overset{n}{\underset{j=1}{\sum }}b_{1j},\overset{n}{\underset{j=1}{\sum }}b_{2j},\ldots ,\overset{n}{\underset{j=1}{\sum }}b_{nj}\right\},\max\left\{\overset{n}{\underset{i=1}{\sum }}b_{i1},\overset{n}{\underset{i=1}{\sum }}b_{i2},\ldots ,\overset{n}{\underset{i=1}{\sum }}b_{in}\right\}\right\}$$

In the next step, the indirect impact ($\hat{B}$) and total impact (T) matrices are determined according to the formulae:

$$\hat{B}=\overset{\infty }{\underset{i=2}{\sum }}BN^{i}\;T=BN+\hat{B}=\overset{\infty }{\underset{i=1}{\sum }}BN^{i}=BN{\left(I-BN\right)}^{-1}$$

Based on matrix elements $T_{n\times n}=\left[t_{ij}\right]$, indicators of significance ($t_{i}^{+}$) are determined describing the overall contribution of the variable in dependencies, as well as indicators of relations ($t_{i}^{-}$), answering the question of whether the variable affects others or is affected by them. The indicators are given in the following formulas:

$$t_{i}^{+}=\overset{n}{\underset{j=1}{\sum }}t_{ij}+\overset{n}{\underset{j=1}{\sum }}t_{ji}\;t_{i}^{+}=\overset{n}{\underset{j=1}{\sum }}t_{ij}-\overset{n}{\underset{j=1}{\sum }}t_{ji}$$

In the DEMATEL method, we used a scale of 0–3 to describe relationships among variables, in which 0—no impact, 1—a small impact, 2—a large impact and 3—a huge impact.

The relationships among social KPI variables are presented in Table 1, while the relationships among economic KPI variables are presented in Table 2.

Step 3. Assessment of a Relative Significance of the KPI Variables

The relative significance of the KPI variables was measured with the DEMATEL method. We propose the use of two basic approaches: indicators of significance ($t^{+}$) and indicators of significance in combination with relationship indicators ($t^{-}$). In the former case, the significance depends on the meaning of the overall role of the variable in the system, while in the latter, the directions of impact are also taken into account.

In the first approach, the higher the value of the significance indicator of the KPI variable, the higher its relative significance, which can be written symbolically as:

$$t_{k}^{+}>t_{l}^{+}\Rightarrow r_{k}^{+}>r_{l}^{+}$$

where:

$t_{i}^{+}$—significance indicator value for the *i* KPI variable,

$r_{i}^{+}$—the rank of the *i* KPI variable determined on the basis of the significance indicator.

We mark this method by $T_{+}$.

In the alternative approach, the value is determined on the basis of the relationship between the functions of the indicators of significance and relationship, which can be written symbolically as:

$$f\left(t_{k}^{+},t_{k}^{-}\right)>f\left(t_{l}^{+},t_{l}^{-}\right)\Rightarrow r_{k}^{+/-}>r_{l}^{+/-}$$

where:

$t_{i}^{-}$—relationship indicator value for the *i* variable included in the KPI,

$r_{i}^{+/-}$—the rank of the *i* KPI variable KPI determined on the basis of significance and relationship indicators.

In this case, we used formulas proposed for DEMATEL by Baykasoglu et al. [27] and Dalalah et al. [28] ($f{\left(t_{i}^{+},t_{i}^{-}\right)}_{}^{BD}$) and Kobryń [29] ($f{\left(t_{i}^{+},t_{i}^{-}\right)}_{}^{K})$ given by:

$$f{\left(t_{i}^{+},t_{i}^{-}\right)}_{}^{BD}=\frac{\sqrt{{\left(t_{i}^{+}\right)}^{2}+{\left(t_{i}^{-}\right)}^{2}}}{\sum_{k=1}^{n}\sqrt{{\left(t_{i}^{+}\right)}^{2}+{\left(t_{i}^{-}\right)}^{2}}}\;f{\left(t_{i}^{+},t_{i}^{-}\right)}_{}^{K}=\frac{\frac{t_{i}^{+}+t_{i}^{-}}{2}}{\sum_{k=1}^{n}\left(\frac{t_{i}^{+}+t_{i}^{-}}{2}\right)}$$

As a consequence,

$$f{\left(t_{k}^{+},t_{k}^{-}\right)}^{K}>f{\left(t_{l}^{+},t_{l}^{-}\right)}^{K}\Rightarrow r_{k}^{K}>r_{l}^{K}$$

and

$$f{\left(t_{k}^{+},t_{k}^{-}\right)}^{BD}>f{\left(t_{l}^{+},t_{l}^{-}\right)}^{BD}\Rightarrow r_{k}^{BD}>r_{l}^{BD}$$

where:

$r_{i}^{k}$—the rank of the *i* KPI variable determined on the basis of indicators of significance and relationship, in accordance with the formula proposed by Kobryn [29] (the method later referred to by K),

$r_{i}^{BD}$—the rank of the *i* KPI variable determined on the basis of indicators of significance and relationship, according to the formula proposed by Baykasoglu et al. [27] and Dalalah et al. [28] (method later referred to by symbol BD).

Step 4. Determination of weights of the KPI variables

The KPI variables weights are based on the hierarchy of significance. In our study, we use well-known weight elicitation methods (formulas cited after Roszkowska [30]).

Rank sum:

$$w_{j}^{RS}\left(R\right)=\frac{2\left(n+1-r_{j}\right)}{n\left(n+1\right)},$$

where:

$w_{j}^{RS}$—weight of the *j* KPI variable determined by the rank sum method,

n—number of KPI variables,

R—rank determination method $R=\{K,BD,T_{+}\}$

$r_{j}$—the rank of the *j* KPI variable determined by method R.

Rank exponent:

$$w_{j}^{RE\left(p\right)}\left(R\right)=\frac{{\left(n+1-r_{j}\right)}^{p}}{\sum_{k=1}^{n}{\left(n+1-r_{k}\right)}^{p}}$$

where:

$w_{j}^{RE\left(p\right)}$—weight of the *j* KPI variable determined by the rank exponent method with parameter p,

n—number of KPI variables,

R—rank determination method $R=\{K,BD,T_{+}\}$

$r_{j}$—the rank of the *j* KPI variable determined by method R.

In this paper, we used parameter *p* in the range of 0.5–3.5 with a step of 0.5. We did not explicitly use *p* = 1, because in this case, the rank exponent weights were equal to the rank sum weights.

Rank reciprocal:

$$w_{j}^{RR}\left(R\right)=\frac{1/r_{j}}{\sum_{k=1}^{n}\left(1/r_{k}\right)}$$

where:

$w_{j}^{RR}$—weight of the *j* KPI variable determined by the rank reciprocal method,

R—rank determination method $R=\{K,BD,T_{+}\}$

$r_{j}$—the rank of the *j* KPI variable determined by method R.

Rank centroid:

$$w_{j}^{ROC}\left(R\right)=\frac{1}{n}\overset{n}{\underset{k=j}{\sum }}\left(1/r_{k}\right)$$

where:

$w_{j}^{ROC}$—weight of the *j* KPI variable determined by the rank centroid method,

R—rank determination method $R=\{K,BD,T_{+}\}$

$r_{j}$—the rank of the *j* KPI variable determined by method R.

The other two sets of weights used in the study were directly given by the functions proposed in [27] and [28] ($f{\left(t_{i}^{+},t_{i}^{-}\right)}_{}^{BD}$) and [29] ($f{\left(t_{i}^{+},t_{i}^{-}\right)}_{}^{K})$, respectively, which we used before to determine the relative significance of the KPI variables. They were given by formulas:

$$w_{j}^{BD}=f{\left(t_{i}^{+},t_{i}^{-}\right)}_{}^{BD}\;w_{j}^{K}=f{\left(t_{i}^{+},t_{i}^{-}\right)}_{}^{K}$$

where:

$t_{i}^{+}$—significance indicator of the *i* KPI variable,

$t_{i}^{-}$—relationship indicator of the *i* KPI variable.

Those two weight elicitation methods used the approach mentioned in the last point, in which weights were ascribed to the *i* variable based on values of both $t_{i}^{+}$ and $t_{i}^{-}$.

It is worth pointing out that the weights did not result from the data, but from the impact assessment carried out with the DEMATEL method. The indirect impacts not visible at first glance were therefore also taken into account.

Step 5. Benchmark determination

Benchmarks were set after converting variables into stimulants. In the dynamic variant, the benchmark was the base situation from period $t_{0}$. In the optimistic and pessimistic variants, we took into account a longer time horizon and used two benchmarks, which were the best ($x^{best}$) and the worst ($x^{worst}$) hypothetical scenario in the period, defined, respectively, as:

$$x^{best}=\left(\underset{t}{\max}x_{1,t}^{\prime },\underset{t}{\max}x_{2,t}^{\prime },\underset{t}{\max}x_{3,t}^{\prime },\underset{t}{\max}x_{4,t}^{\prime },\underset{t}{\max}x_{5,t}^{\prime }\right)\;x^{worst}=\left(\underset{t}{\min}x_{1,t}^{\prime },\underset{t}{\min}x_{2,t}^{\prime },\underset{t}{\min}x_{3,t}^{\prime },\underset{t}{\min}x_{4,t}^{\prime },\underset{t}{\min}x_{5,t}^{\prime }\right)$$

where: $x_{i,t}^{\prime }$—variable value $x_{i}^{\prime }$ in period t.

It is worth mentioning that benchmarks can also be determined in an expert or non-expert manner on the basis of historical data, for example, forecasts of a certain desirable state, planned in the assumptions of economic policy.

Step 6. Calculation of the indicator in relation to the selected benchmark

The calculation of the value of economic indicators in relation to the selected benchmark were carried out according to the following formulae (index E refers to the economic KPI, and the values for sociological indicators were determined in the same way):

1. Dynamic variant:

$$KPI_{t,t_{0}}^{E}=\overset{5}{\underset{i=1}{\sum }}\frac{x_{i,t}^{\prime }}{x_{i,t_{0}}^{\prime }}\cdot 100\cdot w_{i}$$

where:

$w_{i}$—weight determined according to the previously discussed methods, $w_{i}\in \left\{w_{i}^{RE\left(p\right)}\left(R\right),w_{i}^{RS}\left(R\right),w_{i}^{RR}\left(R\right),w_{i}^{ROC}\left(R\right),w_{i}^{BD},w_{i}^{K}\right\}$,

$t_{0}$—base period.

2. Optimistic variant:

$$KPI_{best}^{E}=\overset{5}{\underset{i=1}{\sum }}\frac{x_{i,t}^{\prime }}{\underset{t}{\max}x_{i,t}^{\prime }}\cdot 100\cdot w_{i}$$

3. Pessimistic variant:

$$KPI_{worst}^{E}=\overset{5}{\underset{i=1}{\sum }}\frac{x_{i,t}^{\prime }}{\underset{t}{\min}x_{i,t}^{\prime }}\cdot 100\cdot w_{i}$$

It is obvious that these are the weighted sums of the components that determine the state of the economy. Table 3 contains five types of data whose values are most important for the development of economic growth. Thus, the value of the indicator and its changes show the level of economic development and its changes, i.e., if this value increases, it reflects economic growth.

In all cases, the value with which they are compared to interpret the values of the indicators is 100. The difference between the values of the indicator and 100 means the percentage the value deviates from the benchmark (in order: values from base period $t_{0}$, a hypothetical best variant built on the basis of data, and a hypothetical worst variant built on the basis of data). Values below 100 indicate a relative deterioration of the situation, and values above 100 indicate an improvement. This results from the definition of indicators that $KPI_{best}^{E}\leq 100$ oraz $KPI_{worst}^{E}\geq 100$.

### 2.2. Data

The GUS data for Poland from 2000–2019 were used.

The variables used were:

E1—GDP in current prices;

E2—Average monthly real gross remuneration in the national economy (previous year = 100);

E3—Inflation (consumer price indices, previous year = 100);

E4—Registered unemployment rate (at the end of the year);

E5—budget deficit (millions of zlotys).

Basic descriptive statistics of the variables used to create KPIs are presented in Table 3.

### 2.3. Software

Data preparation and the determination of KPI values is based on the spreadsheet. The programme R was used [31] (version 4.0.2 2020-06-22 “Taking Off Again”, R Core Tea, R Foundation for Statistical Computing, Vienna, Austria) to determine the value of relationship and significance indicators for the DEMATEL matrix. The following packages were also used: corrplot [32] (correlation analysis, company, city and country) and agricolae [33] (median difference tests, company, city and country).

## 3. Results

### 3.1. Significance of Each Variable for the Construction of KPIs

The number 102.5 was assumedas a parameter ci when converting inflation into a stimulant.

The DEMATEL method was used to determine the values of indicators of significance ($t^{+}$) and relationship ($t^{-}$) for each KPI variable. These values are presented in Table 4.

Based on the values of the indicators of significance ($t^{+}$) and relationship ($t^{-}$), the ranks of the significance of the KPI variables were then determined ($r_{j},r_{j}=1,2,\ldots ,5$), where a higher weight corresponds to a higher rank. In the next step, ranks were used to determine the weights of the KPI variables. We used four approaches, described previously in the article. The weights obtained are shown in Table 5.

The weights obtained when using only the indicators of significance (the method described in the previous part of the article with the symbol $T_{+}$) are identical to the weights obtained on the basis of the ranks determined with the BD method, which accounted for indicators $t^{+}$ as well as $t^{-}$. Therefore, in a further part of the article, we will take into account only the results obtained with the BD method. The results indicate that the hierarchy of significance of the KPI variables are highly consistent, and the weights of each variable in different approaches are comparable. For weights based on indicator values $t^{+}$ and $t^{-}$, in the manner proposed by Kobryn [29] (marked with K in this work), a relatively higher significance of remuneration was obtained (for the economic KPI). There are relatively more differences for the sociological KPI. In the BD approach, the feeling of threat is most significant, while in K, the social capital is most significant. The position of the criterion “sense of wealth” is also clearly changing, and is third in the hierarchy determined on the basis of BD ranks, while in the case of K, it is last. The average positions of the other two criteria, i.e., the system assessment and the sense of monetary availability, are also different.

### 3.2. Dynamic Indicators ($KPI_{t,t_{0}}^{E}$)

In the next step, we determined the values of KPI economic indicators. First, let us present the results obtained in the dynamic approach ($KPI_{t,t_{0}}^{E}$), in which we used the previous year’s value ($t_{0}=t-1$ as the base value.

**Figure 2:**
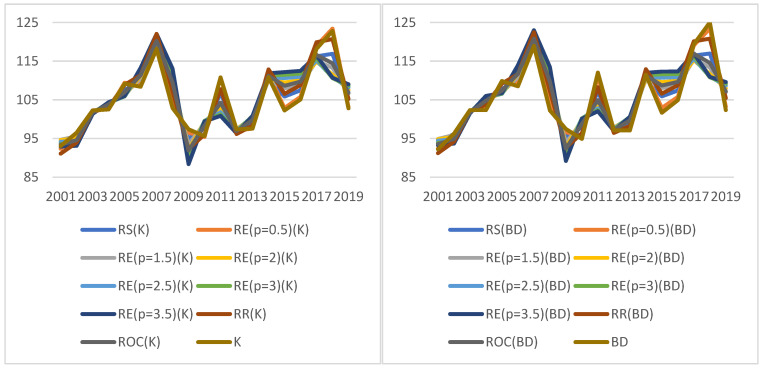
Values of indicators for economic KPIs ($KPI_{t,t_{0}}^{E}$). Source: authors’ own material based on the GUS data.

The values of the indicators for the economic KPI and the determined weights are shown in Figure 2.

The graphs show the value of the dynamic KPI^E^ in the selected period for two calculation methods (K—the graph on the left, and BD—the graph on the right) as well as 10 weights for each method. It turns out that the indicator takes similar values regardless of the method and weights adopted. It proves the stability of the results obtained, which means reliability of the basis for decision making.

**Figure 3:**
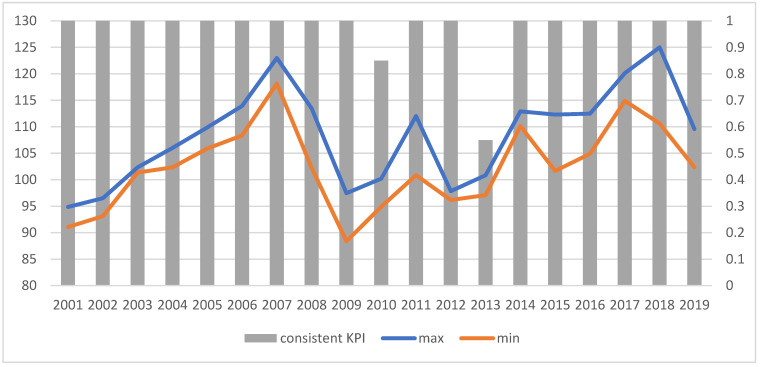
The range of indicator values (left axis) and consistency of results (right axis) for $KPI_{t,t_{0}}^{E}$. “Consistent KPIs”—KPIs showing the same direction of change compared to the previous year. Source: authors’ own material based on the GUS data.

It can be easily seen that the indicators are formed according to the same general trends, regardless of the approach chosen to construct them. The discrepancies in the values of the indicators are not high (the ratio of the maximum to the minimum value does not exceed 1.14, and its average value measured by the median was 1.0455). The range of the indicator values fluctuates. The indicators are very much consistent with the conclusions on the improvement or deterioration of the situation. They all usually indicate the same direction of change, and only in 2010 and 2013 can some discrepancies be observed; three and nine indicators, respectively, indicate improvement in the situation (compared to the previous year), while the others indicate deterioration (Figure 3).

The average KPIs determined by the BD method are almost always (except for 2013 and 2016) higher than those determined by the K method. In both years, this applies to all methods of determining weights. In addition, the KPI median values determined by the K and BD methods were compared (Table 6). It can be noted that, relatively, the smallest difference between the median values of the indicators calculated by the K method and BD occurs for weights determined by the RS method (0.047%), the largest for weights determined by the RE method with parameter *p* = 3.5 (0.716%). These are not big differences. The differences in median levels are not statistically significant for a significance level of 0.05.

### 3.3. Optimistic Indicators ($KPI_{best}^{E}$)

**Figure 4:**
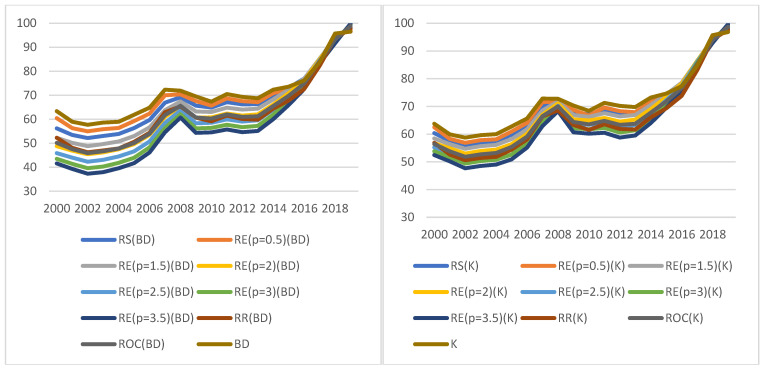
Indicator values for the economic KPI ($KPI_{best}^{E}$). Source: authors’ own material based on the GUS data.

In the case of optimistic indicators ($KPI_{best}^{E}$), the conclusions are similar. The indicators follow the same general trends regardless of the approach chosen to construct them, and the differences between heights determined on the basis of different weight values decline over time (Figure 4). Again, regardless of the method and weights adopted, analogous values are obtained for the optimistic KPI^E^, which proves the stability of results, and thus the reliability of the basis for decision making.

**Figure 5:**
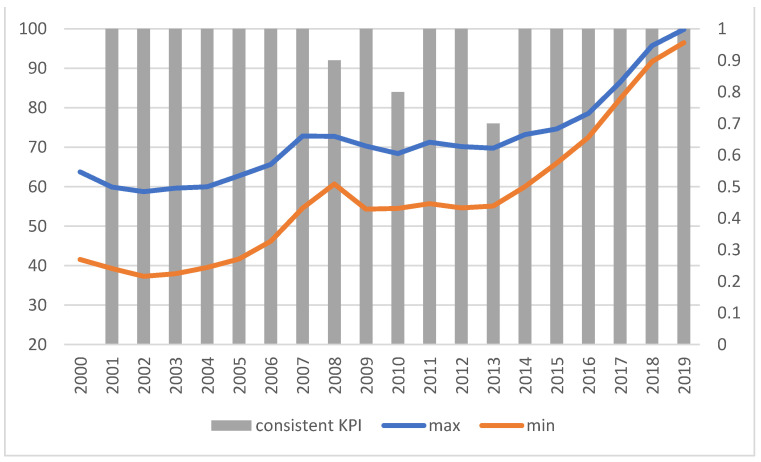
The range of indicator values (left axis) and consistency of results (right axis) for $KPI_{best}^{E}$. “Consistent KPIs”—KPIs showing the same direction of change compared to the previous year. Source: authors’ own material based on the GUS data.

The relation of the maximum to the minimum value exceeds 1.5, and its average value measured by the median is 1.2822. The indicators are quite consistent with the conclusions on the improvement or deterioration of the situation. They all usually indicate the same direction of change; only in 2008, 2010 and 2013, can some discrepancies observed: 18, 4 and 14 indicators, respectively, indicated some improvement in the situation, while the others indicated deterioration (Figure 5).

As shown in Table 7, the differences between the maximum and minimum average KPIs, measured by medians, do not exceed 29%. The smallest differences in KPI values between the approaches using BD and K ranks occur for the variant without weights, and the smallest for weights determined by the RE method (with parameter *p* = 3.5) RR. These differences do not exceed 11%. Differences in median levels are not statistically significant for a significance level of 0.05.

### 3.4. Pessimistic Indicators ($KPI_{worst}^{E}$)

**Figure 6:**
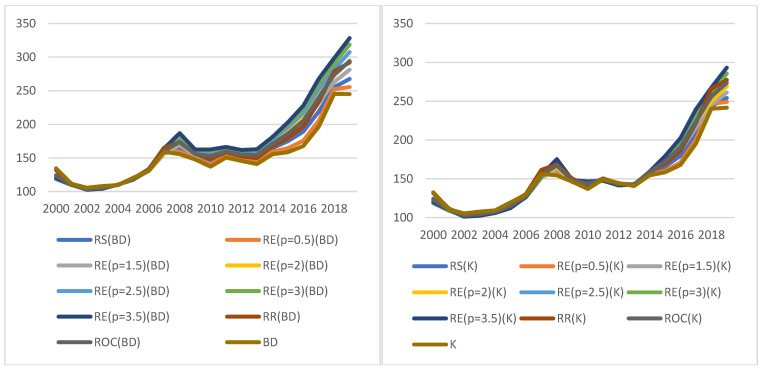
Values of indicators for economic KPI ($KPI_{worst}^{E}$). Source: authors’ own material based on the GUS data.

In the case of pessimistic indicators ($KPI_{worst}^{E}$), similar conclusions can be drawn as in the previous variants. As shown in Figure 6, again, the indicators follow the same general trends regardless of the approach chosen to construct them, but this time the gap between volumes determined on the basis of different weight values increases over time. Furthermore, once more, regardless of the method and weights adopted, analogous values are obtained for the pessimistic KPI^E^, which indicates the stability of the results, and thus the reliability of the basis for decisions making.

**Figure 7:**
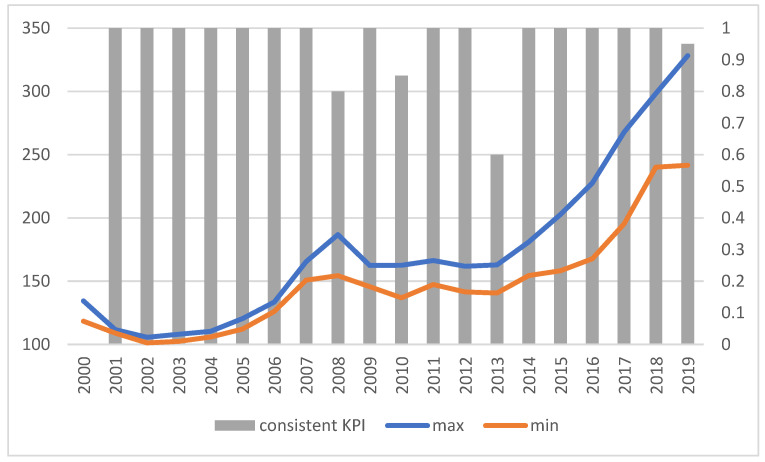
The range of indicator values (left axis) and consistency of results (right axis) for $KPI_{worst}^{E}$.“Consistent KPIs”—KPIs showing the same direction of change compared to the previous year. Source: authors’ own material based on the GUS data.

The ratio of the maximum and minimum value does not exceed 1.37; on average, it is 1.2114 (median). As shown in Figure 7, the values of the indicators are relatively consistent with the direction of changes (improvement or deterioration of the indicator value over the previous year), although there are divergent conclusions (for 3 years).

The differences between the maximum and minimum average KPIs, measured by medians, do not exceed 13%. The smallest differences in KPI values between approaches using BD and K ranks occur for weights determined by the RE method (with parameter *p* = 3.5), and the smallest for the case not accounting for weights (Table 8). The differences in median levels are not statistically significant for a significance level of 0.05.

## 4. Discussion

The indicator values were compared with each other. To this end, all of the time series were limited to the years 2001–2019 to ensure their equal length, and the series were then standardised using the formula:

$$KPI_{t}^{\prime }=\frac{KPI_{t}}{\underset{t}{\max}KPI_{t}},$$

where: $KPI_{t}$—the initial KPI value over period t,

$KPI_{t}^{\prime }$—the normalised KPI value over period t.

The obtained values belong to the range (0−1).

In the next step, we examined the similarities in the formation of time series using, for this purpose, the correlation analysis and Euclidean distance test.

The distance between time series is calculated from the formula:

$$d\left({KPI}_{1}^{\prime },{KPI}_{2}^{\prime }\right)=\sqrt{\sum_{t=1}^{19}{\left({KPI}_{1,t}^{\prime }-{KPI}_{2,t}^{\prime }\right)}^{2}},$$

where:

$KPI_{i,t}$—the normalised value *i* of this KPI indicator in period t.

**Figure 8:**
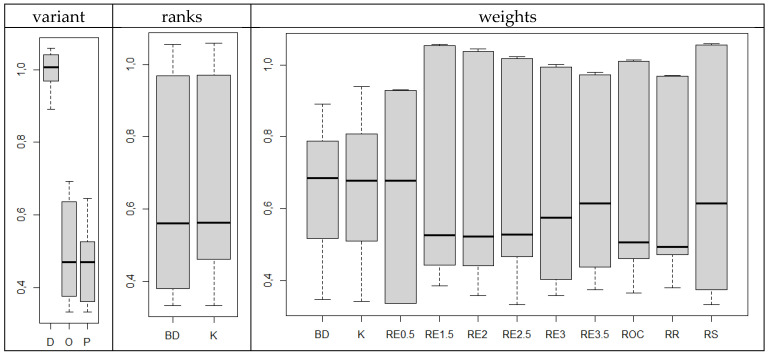
Distance distributions *d* $\left({KPI}_{1}^{\prime },{KPI}_{2}^{\prime }\right)$ according to the approaches used in the construction of indicators. Source: authors’ own material based on the GUS data.

The Euclidean distance test of the values of standardised indicators allows us to distinguish approaches for which the obtained results are the closest to the others, as well as which are the most different. The distance distributions calculated for the series, presented in Figure 8, indicate that, on average, the smallest distances are characteristic of KPIs determined by an optimistic approach (${{KPI}^{\prime }}_{best}^{E}$) and pessimistic (${{KPI}^{\prime }}_{worst}^{E}$) (which, in our approach, is equivalent to the greatest similarity to the other series). In addition, on average, smaller distances are characteristic of the indicators for the construction of which the ranks determined by the BD method, rather than K, were used. The weights determined in the BD approach also have the greatest average similarity to other methods.

The analysis of the statistical significance of median differences indicates that statistically significant differences occur only in the first case; i.e., medians do not differ significantly due to the method of determining ranks or weights, but optimistic and pessimistic approaches are (with a significance level of 0.05) significantly different from the dynamic approach (*p*-value is 0 in both cases), while not being significantly different from each other. It can, therefore, be considered that the indicators $KPI_{best}^{E}$ and $KPI_{worst}^{E}$ are better representatives of all discussed types of KPI than $KPI_{t,t_{0}}^{E}$.

Next, we examined the similarity of individual time series with the correlation analysis of their values. The Pearson correlation coefficient, given in a formula, as well as the Spearman rank correlation coefficient, were used. The use of the latter was due to the fact that the similarity of the KPI series can also be reflected in the reciprocal value ratio.

**Figure 9:**
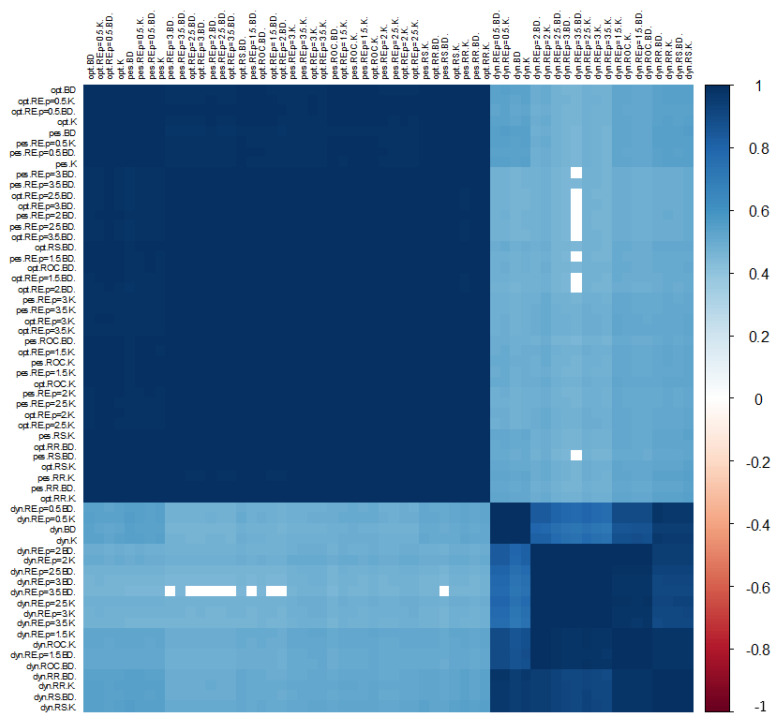
Illustration of the value of Pearson correlation coefficients for KPIs by the approaches used in the construction of indicators. Source: authors’ own material based on the GUS data.

The results of the analysis of the Pearson correlation coefficient are presented in Figure 9, where empty fields indicate statistically insignificant values at a significance level of 0.05. The minimum value of the Pearson correlation coefficient obtained is 0.4532, while the average value is 0.9731. A considerable majority of KPIs were characterised by a significant, high similarity. This is especially true of the indicators $KPI_{best}^{E}$ and $KPI_{worst}^{E}$, which are the closest to each other. $KPI_{t,t_{0}}^{E}$ is, relatively, the most different. Thus, the indicators in the dark fields behave in the same way, so it is possible to choose the indicator that is most universal.

**Figure 10:**
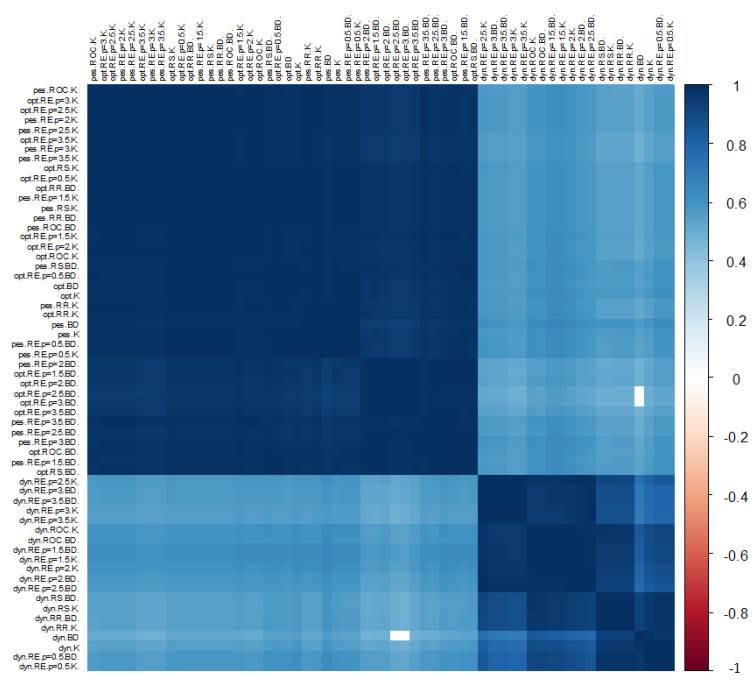
Illustration of the value of Spearman rank correlation coefficients for KPIs by the approaches used in the construction of indicators. Source: authors’ own material based on the GUS data.

The results obtained for the Spearman rank correlation coefficient are presented in Figure 10. Almost all of the KPIs exhibit statistically significant similarity. The minimum value of the correlation coefficient is 0.4439, while the average is 0.95. The conclusions on the similarity of the approaches confirm those drawn before. The indicators $KPI_{best}^{E}$ and $KPI_{worst}^{E}$ are again grouped as the most similar. Similarly, in this case, the indicators in the dark fields behave in the same way, so it is possible to choose the one that is the most universal.

It can, therefore, be concluded that the choice of the method of rank determination and weighting components is secondary to the approach in question. It is important to choose between dynamic, optimistic and pessimistic variants. The indicators $KPI_{best}^{E}$ and $KPI_{worst}^{E}$ seem to be universal, and can represent others.

## 5. Conclusions

In the present article, the authors focus on the most important problem faced by health systems in the world. In the face of hard budget constraints and an increasingly conspicuous shortage of money, the achievement of the fundamental goal of clinical and economic efficiency has become a considerable problem. A way of financing healthcare providers in which experts see an opportunity to improve the situation is VBHC. As demonstrated in the literature review, it is a very complex way of funding and requires some interdisciplinary cooperation. The issues of coordination, integration, measurement of healthcare results and the cost of achieving them, as well as payments for medical technologies, have been a subject of research for quite some time. Thus, the inclusion of personalised medicine in selected fields of medicine also seems obvious. However, an issue that has recently been a challenge for researchers is indicators. When it comes to KPIs for hospitals, much has already been done. However, the very operation of hospitals is a resultant of all determinants, i.e., social behaviour, the state of the economy, public finances and the healthcare system. Since the subject of the impact of the state of the economy and sociological factors has, so far, been very rarely discussed, this article focuses on them. Based on the literature, theoretical and empirical achievements concerning the importance of KPIs for the effective functioning of health care in the PM-based VMHC model are presented. That is why it is so important to create KPIs that provide knowledge about all of the determinants of achievements/failures in health care. On the example of economic KPIs calculated for selected quantitative data for Poland, it was shown how knowledge about the basis for making decisions increases. As the article was written during the pandemic and, unfortunately, due to the changes in society’s views on their situation, which are likely to have resulted from the COVID-19 outbreak, it was not possible to conduct adequate surveys; thus the presentation of the issue of social impacts is based on the possessed possibilities. However, the entire analytical process was presented for economic determinants using macroeconomic data. The economic KPIs were constructed on the basis of the five most important data that affect the level of development and growth of the economy. Dynamic, optimistic and pessimistic indicators were presented. Their statistical analysis was carried out, which showed statistical significance, and most importantly, the stability of the economic values of KPIs. This means that the state of the economy, i.e., the state of public finances based on it (though not exclusively) and the financing of healthcare, constitute a solid basis for making decisions concerning healthcare stakeholders. The obtained results for Poland in the analysed period show that optimistic or pessimistic KPIs should be used, and in particular, that they give similar results, which confirms the reliability of the method to be used in the decision-making process. A study focused on the survey results and sociological KPI is planned as a possible future line of research.

## Figures and Tables

**Table 1:** Relationships among the social KPI variables.

		S1	S2	S3	S4	S5
		System Assessment	Sense of Monetary Availability	“Social Capital”	Sense of Threat	Sense of Wealth
S1	system assessment	0	1	2	3	2
S2	sense of monetary availability	0	0	2	3	3
S3	“social capital”	3	2	0	3	2
S4	sense of threat	3	2	2	0	2
S5	sense of wealth	1	3	1	3	0

**Table 2:** Relationships among the economic KPI variables.

	GDP	Remuneration	Inflation	Employment	Budget Deficit
GDP	0	2	1	3	2
remuneration	2	0	3	2	1
inflation	2	2	0	2	1
employment	3	3	1	0	1
budget deficit	1	0	2	1	0

**Table 3:** Basic descriptive statistics of variables used to create economic KPIs.

		Min	Q1	Q2	Mean	Q3	Max	SD
E1	GDP	748,483	976,170	1409,435	1,404,239	1,733,711	2,287,738	474,686.4
E2	Remuneration	100.1	101.4	103	103	104.3	105.9	1.778372
E3	Inflation (previous year = 100)	99.1	101	102.2	102.5	103.5	110.1	2.439046
E4	Registered unemployment rate (%)	5.2	9.65	12.45	12.77	15.7	20	4.52689
E5	Deficit	10,406	24,221	28,669	29,637	39,906	46,160	10,895.31

Q1—quartile one, Q2—median, Q3—quartile three, SD—standard deviation. Source: authors’ own material based on the GUS data.

**Table 4:** Values of indicators of significance ($t^{+}$ ) and relationship ($t^{-}$ ) for each KPI variable.

**Sociological**					
	**S1**	**S2**	**S3**	**S4**	**S5**
	**System Assessment**	**Sense of Monetary Availability**	**“Social Capital”**	**Sense of Threat**	**Sense of Wealth**
$t^{+}$	4.557	4.801	4.997	5.948	4.980
$t^{-}$	0.230	−0.019	0.760	−0.709	−0.262
**Economic**					
	**E1**	**E2**	**E3**	**E4**	**E5**
	**GDP**	**Remuneration**	**Inflation**	**Employment**	**Budget Deficit**
$t^{+}$	19.871	19.370	17.60061	20.308	11.899
$t^{-}$	−0.225	0.799	0.552995	0.212	−1.339

**Table 5:** Weights of KPI variables.

	Sociological KPI					Economic KPI				
	S1 System Assessment	S2 Sense of Monetary Availability	S3 “Social Capital”	S4 Sense of Threat	S5 Sense of Wealth	E1 GDP	E2 Remuneration	E3 Inflation	E4 Employment	E5 Budget Deficit
**RS** $w_{j}^{RS}\left(BD\right)$	0.0667	0.1333	0.2667	0.3333	0.2000	0.2667	0.2000	0.1333	0.3333	0.066667
**RE(0.5)** $w_{j}^{RE\left(p=0.5\right)}\left(BD\right)$	0.1193	0.1687	0.2386	0.2668	0.2066	0.2386	0.2066	0.1687	0.2668	0.119299
**RE(1.5)** $w_{j}^{RE\left(p=1.5\right)}\left(BD\right)$	0.0355	0.1003	0.2836	0.3964	0.1842	0.2836	0.1842	0.1003	0.3964	0.035455
**RE(2)** $w_{j}^{RE\left(p=2\right)}\left(BD\right)$	0.0182	0.0727	0.2909	0.4545	0.1636	0.2909	0.1636	0.0727	0.4545	0.018182
**RE(2.5)** $w_{j}^{RE\left(p=2.5\right)}\left(BD\right)$	0.0091	0.0514	0.2905	0.5075	0.1415	0.2905	0.1415	0.0514	0.5075	0.009079
**RE(3)** $w_{j}^{RE\left(p=3\right)}\left(BD\right)$	0.0044	0.0356	0.2844	0.5556	0.1200	0.2844	0.1200	0.0356	0.5556	0.004444
**RE(3.5)** $w_{j}^{RE\left(p=3.5\right)}\left(BD\right)$	0.0021	0.0242	0.2743	0.5990	0.1002	0.2743	0.1002	0.0242	0.5990	0.002143
**RR** $w_{j}^{RR}\left(BD\right)$	0.0876	0.1095	0.2190	0.4380	0.1460	0.2190	0.1460	0.1095	0.4380	0.087591
**ROC** $w_{j}^{ROC}\left(BD\right)$	0.0400	0.0900	0.2567	0.4567	0.1567	0.2567	0.1567	0.0900	0.4567	0.04
**RS** $w_{j}^{RS}\left(K\right)$	0.2000	0.1333	0.3333	0.2667	0.0667	0.2000	0.2667	0.1333	0.3333	0.066667
**RE(0.5)** $w_{j}^{RE\left(p=0.5\right)}\left(K\right)$	0.2066	0.1687	0.2668	0.2386	0.1193	0.2066	0.2386	0.1687	0.2668	0.119299
**RE(1.5)** $w_{j}^{RE\left(p=1.5\right)}\left(K\right)$	0.1842	0.1003	0.3964	0.2836	0.0355	0.1842	0.2836	0.1003	0.3964	0.035455
**RE(2)** $w_{j}^{RE\left(p=2\right)}\left(K\right)$	0.1636	0.0727	0.4545	0.2909	0.0182	0.1636	0.2909	0.0727	0.4545	0.018182
**RE(2.5)** $w_{j}^{RE\left(p=2.5\right)}\left(K\right)$	0.1415	0.0514	0.5075	0.2905	0.0091	0.1415	0.2905	0.0514	0.5075	0.009079
**RE(3)** $w_{j}^{RE\left(p=3\right)}\left(K\right)$	0.1200	0.0356	0.5556	0.2844	0.0044	0.1200	0.2844	0.0356	0.5556	0.004444
**RE(3.5)** $w_{j}^{RE\left(p=3.5\right)}\left(K\right)$	0.1002	0.0242	0.5990	0.2743	0.0021	0.1002	0.2743	0.0242	0.5990	0.002143
**RR** $w_{j}^{RR}\left(BK\right)$	0.1460	0.1095	0.4380	0.2190	0.0876	0.1460	0.2190	0.1095	0.4380	0.087591
**ROC** $w_{j}^{ROC}\left(K\right)$	0.1567	0.0900	0.4567	0.2567	0.0400	0.1567	0.2567	0.0900	0.4567	0.04
$w_{j}^{BD}$	0.1795	0.1891	0.1991	0.2359	0.1964	0.2229	0.2175	0.1975	0.2278	0.13431
$w_{j}^{K}$	0.1894	0.1891	0.2277	0.2072	0.1866	0.2206	0.2265	0.2039	0.2304	0.119
**RS** $w_{j}^{RS}(T_{+})$	0.0667	0.1333	0.2667	0.3333	0.2000	0.2667	0.2000	0.1333	0.3333	0.066667
**RE(0.5)** $w_{j}^{RE\left(p=0.5\right)}(T_{+})$	0.1193	0.1687	0.2386	0.2668	0.2066	0.2386	0.2066	0.1687	0.2668	0.119299
**RE(1.5)** $w_{j}^{RE\left(p=1.5\right)}(T_{+})$	0.0355	0.1003	0.2836	0.3964	0.1842	0.2836	0.1842	0.1003	0.3964	0.035455
**RE(2)** $w_{j}^{RE\left(p=2\right)}(T_{+})$	0.0182	0.0727	0.2909	0.4545	0.1636	0.2909	0.1636	0.0727	0.4545	0.018182
**RE(2.5)** $w_{j}^{RE\left(p=2.5\right)}(T_{+})$	0.0091	0.0514	0.2905	0.5075	0.1415	0.2905	0.1415	0.0514	0.5075	0.009079
**RE(3)** $w_{j}^{RE\left(p=3\right)}(T_{+})$	0.0044	0.0356	0.2844	0.5556	0.1200	0.2844	0.1200	0.0356	0.5556	0.004444
**RE(3.5)** $w_{j}^{RE\left(p=3.5\right)}(T_{+})$	0.0021	0.0242	0.2743	0.5990	0.1002	0.2743	0.1002	0.0242	0.5990	0.002143
**RR** $w_{j}^{RR}(T_{+})$	0.0876	0.1095	0.2190	0.4380	0.1460	0.2190	0.1460	0.1095	0.4380	0.087591
**ROC** $w_{j}^{ROC}(T_{+})$	0.0400	0.0900	0.2567	0.4567	0.1567	0.2567	0.1567	0.0900	0.4567	0.04

**Table 6:** Economic KPI medians ($KPI_{t,t_{0}}^{E}$ ) determined by methods K and BD.

	BD	K
RS	105.8897	105.8396
RE (*p* = 0.5)	103.2233	103.1181
RE (*p* = 1.5)	106.9213	106.4883
RE (*p* = 2)	106.5803	106.026
RE (*p* = 2.5)	106.528	105.879
RE (*p* = 3)	106.6115	105.8952
RE (*p* = 3.5)	106.7471	105.9887
RR	106.5819	106.527
ROC	107.1898	106.8607
bez wag	102.3405	102.7958

Source: authors’ own material based on the GUS data.

**Table 7:** Economic KPI medians ($KPI_{best}^{E}$ ) determined by methods K and BD.

	BD	K
RS	66.2236	68.0211
RE (*p* = 0.5)	67.5448	68.5072
RE (*p* = 1.5)	63.8937	66.8734
RE (*p* = 2)	61.2202	65.2883
RE (*p* = 2.5)	58.7663	63.5575
RE (*p* = 3)	56.5474	61.9635
RE (*p* = 3.5)	54.5901	60.3429
RR	60.3829	62.5806
ROC	61.0858	64.0558
no weights	69.395	70.2309

Source: authors’ own material based on the GUS data.

**Table 8:** Medians of economic KPI ($KPI_{worst}^{E}$ ) determined by methods K and BD.

	BD	K
RS	151.6572	145.0469
RE (*p* = 0.5)	149.0889	145.9193
RE (*p* = 1.5)	155.0524	144.6197
RE (*p* = 2)	157.7133	145.0635
RE (*p* = 2.5)	159.7628	145.8747
RE (*p* = 3)	161.566	146.6294
RE (*p* = 3.5)	162.742	147.0796
RR	154.1955	146.9581
ROC	155.4681	145.705
Without weights	146.569	145.3966

Source: authors’ own material based on the GUS data.

## Data Availability

The data are publicly available.
